# Supplementary material for: Two-dimensional optomechanical crystal cavity with high quantum cooperativity
Source: Nat Commun. 2020 Jul 6;11:3373. doi: 10.1038/s41467-020-17182-9 (PMC7338352; doi:10.1038/s41467-020-17182-9)
Supplement: Supplementary file 1 — Supplementary Information [file 41467_2020_17182_MOESM1_ESM.pdf]

Supplementary Information for: 'Two-Dimensional Optomechanical Crystal Cavity With High Quantum Cooperativity'

Hengjiang Ren,[1,2,3] Matthew H. Matheny,[1,2,3] Gregory S. MacCabe,[1,2,3] Jie Luo,[1,2,3] Hannes Pfeifer,[4,*] Mohammad Mirhosseini,[1,2,3] and Oskar Painter[1,2,3,†]

[1] *Thomas J. Watson, Sr., Laboratory of Applied Physics, California Institute of Technology, Pasadena, California 91125, USA*
[2] *Kavli Nanoscience Institute, California Institute of Technology, Pasadena, California 91125, USA*
[3] *Institute for Quantum Information and Matter, California Institute of Technology, Pasadena, California 91125, USA*
[4] *Max Planck Institute for the Science of Light, Staudtstrasse 2, 91058 Erlangen, Germany*

(Dated: May 17, 2020)

* Current Address: Institut für Angewandte Physik, Universität Bonn, Wegelerstraße 8, 53115 Bonn, Germany
† opainter@caltech.edu; http://copilot.caltech.edu

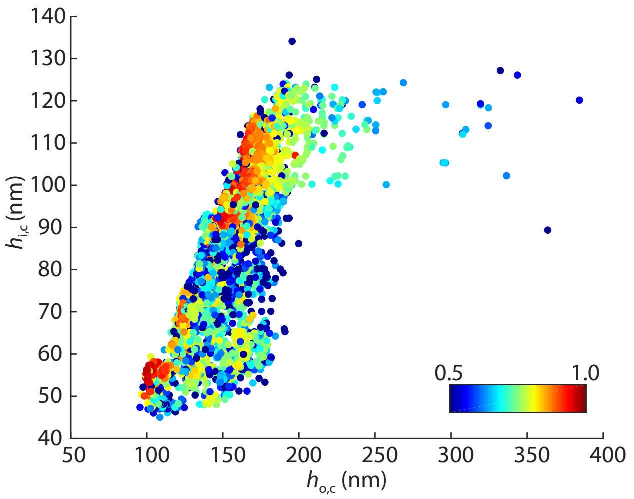

FIG. S-1. **Nelder-Mead simplex search pattern.** A slice of the multidimensional parameter space explored by the Nelder-Mead minimization method. The color of the points indicate the normalized value of the fitness function. This slice includes multiple Nelder-Mead search runs with randomly generated starting points and convergence to multiple hot-spots in the two-dimensional space of $h_{\mathrm{i,c}}$ and $h_{\mathrm{o,c}}$.

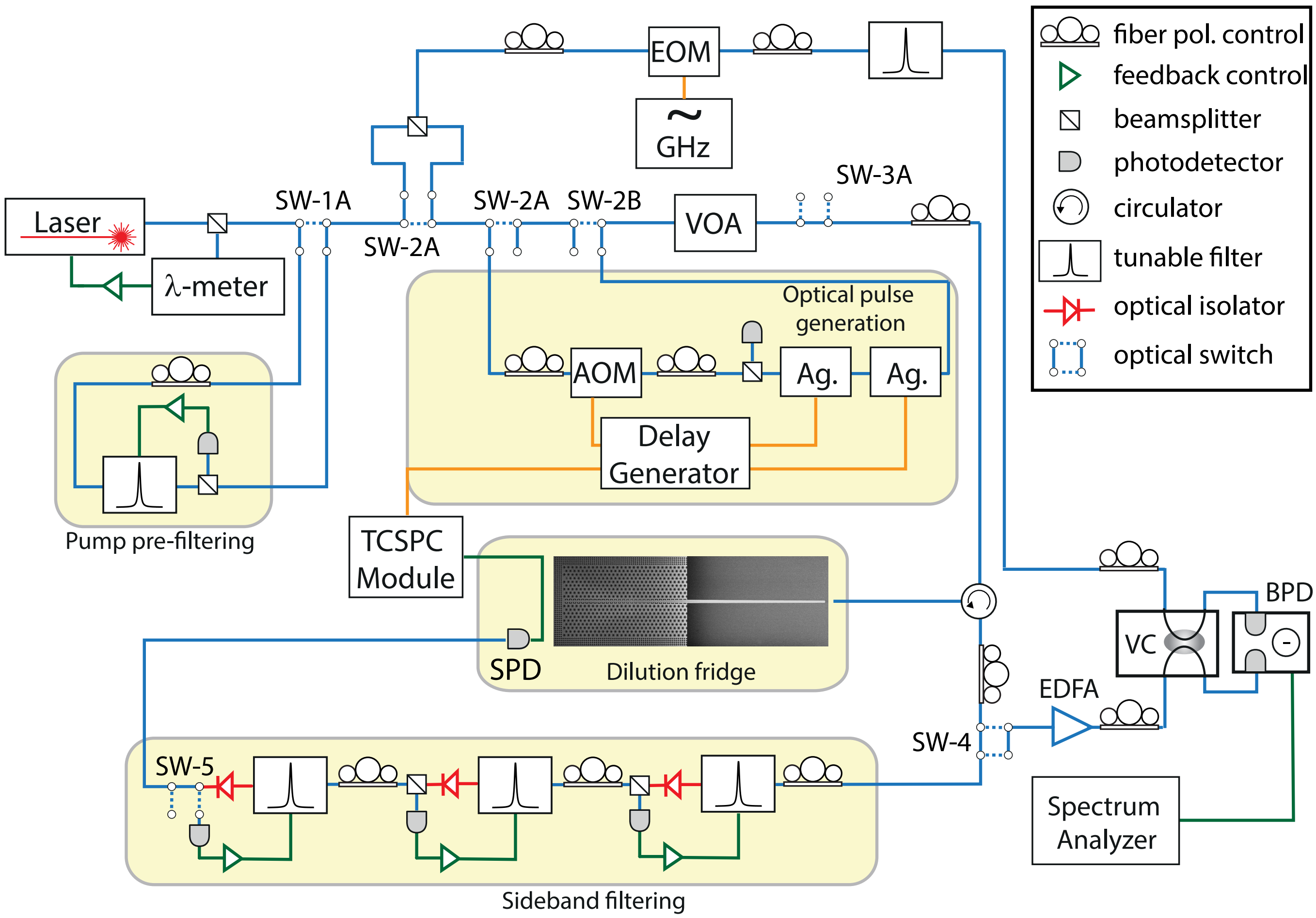

FIG. S-2. **Diagram of the experimental setup.** A 1550nm external cavity tunable laser is used to generate the optical pump signal in this work. The laser is initially passed through a single 50 MHz-bandwidth filter to suppress broadband spontaneous emission noise, after which it can be switched between two different paths: (i) a heterodyne spectroscopy path, and (ii) a photon counting path. In the photon counting path, an acousto-optic modulator (AOM) in series with a fast switch (Ag.) is used for generating high-extinction optical pulses. The modulation components are triggered by a digital delay generator. In the heterodyne spectroscopy path, the light is divided into two paths, one path is passed through an electro-optic intensity modulator (EOM) and a filter to generate the local oscillator (LO) signal, the other path is sent to the optomechanical device. Upon reflection from the device under test, a circulator routes the reflected laser light to either: (i) an EDFA, tunable variable optical coupler (VC), balanced photodiodes (BPD) and spectrum analyzer, or (ii) a sideband-filtering bank consisting of three cascaded fiber Fabry-Perot filters (Micron Optics FFP-TF2) and a SPD operated at 760 mK. λ-meter: wavemeter, EOM: electro-optic intensity modulator, AOM: acousto-optic modulator, Ag.: Agiltron 1x1 MEMS switch, SW: optical 2×2 switch, VOA: variable optical attenuator, EDFA: erbium-doped fiber amplifier, BPD: balanced photodetector, SPD: single photon detector, TCSPC: time-correlated single photon counting module (PicoQuant PicoHarp 300).

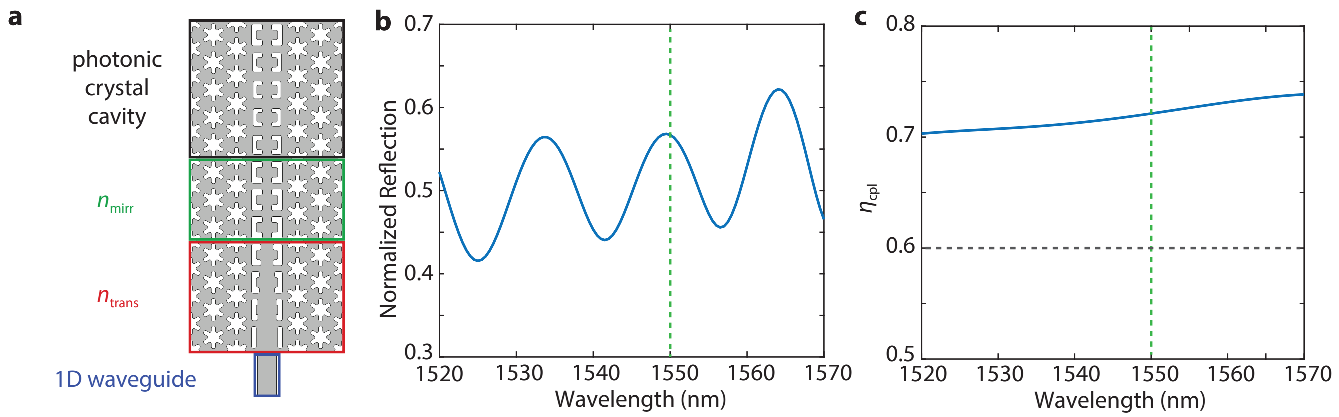

FIG. S-3. **Optical coupling to devices.** **a**, Schematic shows the design of the full quasi-2D snowflake OMC device, including central OMC cavity region (black), front mirror section of the OMC cavity (green), OMC cavity to line-defect waveguide transition region (red), and tapered coupling waveguide (blue). **b**, Broadband reflection spectrum of the optimized coupling waveguide design. Dashed vertical line is the nominal operating wavelength. **c**, Zoom-in of the coupling curve around the operational point, where the blue solid curve is the simulated coupling level and the dashed grey curve is the measured single-pass coupling efficiency level, η_{cpl}.

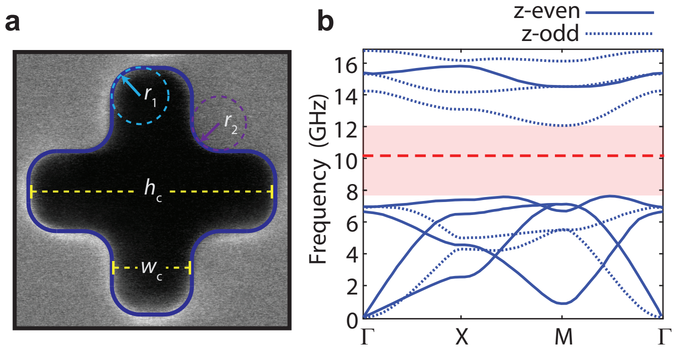

FIG. S-4. **Cross-structure acoustic bandgap shield.** **a**, SEM image of an individual unit cell of the cross-crystal acoustic shield. The dashed lines show fitted geometric parameters used in simulation, including cross height (h_c = 223 nm), cross width (w_c = 75 nm), inner fillet radius (r_1 = 35 nm), and outer fillet radius (r_2 = 35 nm). Thickness of silicon device layer is 220 nm. **b**, Bandstructure of the realized cross-crystal shield unit cell, with the full bandgap highlighted in pink. Solid (dotted) lines correspond to modes of even (odd) symmetry in the direction normal to the plane of the unit cell. The dashed red line indicates the mechanical breathing-mode frequency at $\omega_m/2\pi = 10.27$ GHz.

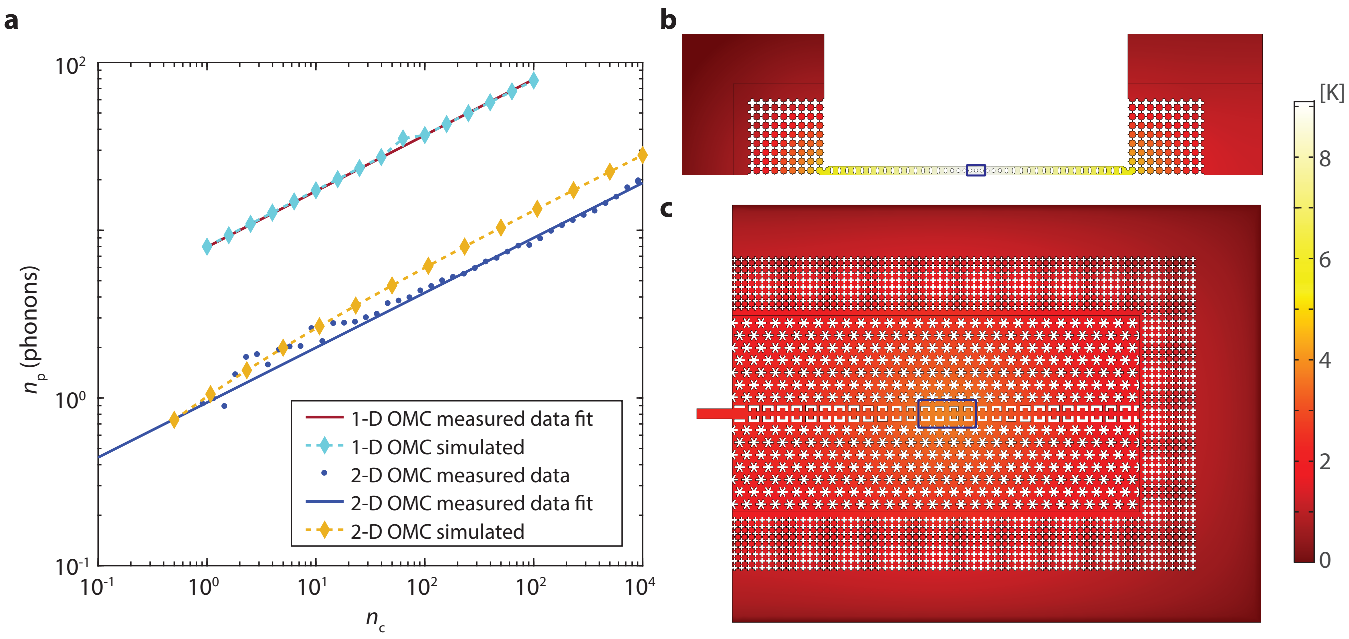

FIG. S-5. **FEM modelling of thermal conductance.** **a**, FEM-simulated, measured and fitted curves of n_{p} for both a 1D nanobeam OMC cavity and a quasi-2D OMC cavity versus number of intra-cavity photons n_{c}. Dashed lines are simulated data and solid lines are fitted curves to measured data. To obtain the material properties used in the simulation, we fit the 1D nanobeam measurement data to a phenomenological thermal conductance model described in the text. The material properties determined from the simulation of the 1D nanobeam were then used to simulate n_{p} versus n_{c} for the quasi-2D OMC cavity. **b**, FEM-simulated temperature profile of the 1D nanobeam OMC cavity. **c**, FEM-simulated temperature profile of the quasi-2D OMC cavity. In both **b** and **c** the intra-cavity photon number is $n_{\mathrm{c}} = 100$ and the temperature scalebar is plotted on the right. The area indicated by blue boxes in the center of both OMC cavities are the heat source used in FEM simulations, where the size the boxes are on the order of optical volume of cavity mode, and total heating power within the boxes volume is P_{th}. The size of the geometries in **b** and **c** are not shown on the same scale.

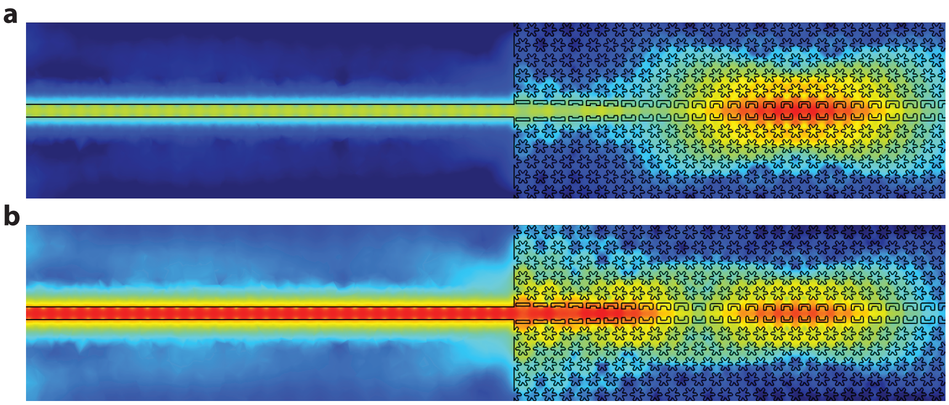

FIG. S-6. **Simulated optical energy density.** Plot of the FEM-simulated time averaged optical field energy density of the quasi-2D OMC cavity with coupling waveguide for **a**, $\Delta = 0$ GHz and **b**, $\Delta = 10$ GHz. Both plots are plotted in logarithm scale and normalized to maximum energy density in each simulation.

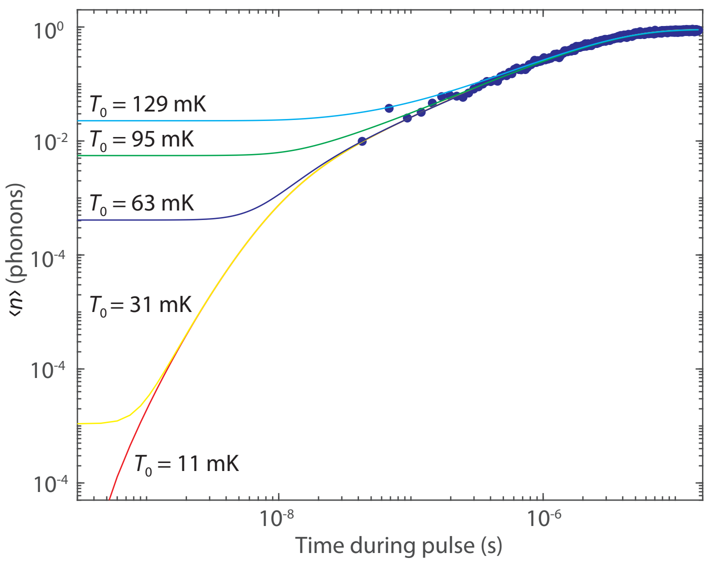

FIG. S-7. **Acoustic mode occupancy at base temperature.** Plot of the measured (filled blue circles) occupancy of the quasi-2D OMC phonon mode at an applied DR temperature of $T_\mathrm{f} \sim 10$ mK as a function of time within the read-out pulse. Here the read-out photon number is chosen to be small ($n_\mathrm{c} = 9.9$) to minimize parasitic heating during the initial time bins of the pulse. Other measurement parameters are $\tau_\mathrm{pulse} = 10$ μs, $\tau_\mathrm{off} = 240$ μs, and measurement photon-counting bin size $\tau_\mathrm{bin} = 25.6$ ns. This measurement is performed on a zero-shield device with parameters (κ, κ_e, g_0, ω_m, γ_0) $= 2\pi$(1.11 GHz, 455 MHz, 1.18 MHz, 10.238 GHz, 21.8 kHz). Calculated curves based upon a phenomenological heating model [1] are shown for $T_0 = 11$ mK (red), 31 mK (yellow), 63 mK (blue), 95 mK (green), 129 mK (cyan) are also plotted for reference.

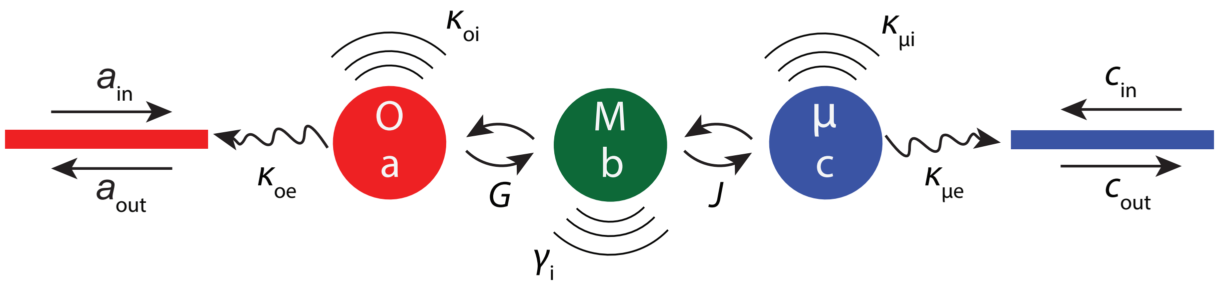

FIG. S-8. **Schematic of bi-directional microwave-to-optics transducer.** Mode-O, mode-M, and mode-μ represent the optical mode, mechanical mode, and microwave mode, respectively. Optical decay rate κ_{oi}, mechanical decay rate γ_{i} and the microwave decay rate $\kappa_{\mu\mathrm{i}}$ are shown. The optomechanical parametrically enhanced coupling rate due to a laser drive is given by $G = g_0\sqrt{n_{\mathrm{c}}}$, where n_{c} is the number of intra-cavity photons in the optical cavity due to the laser drive.

1. CAVITY DESIGN OPTIMIZATION

Using a Finite-Element Method (FEM), we simulated the OMC cavity geometry to determine the optical and mechanical cavity mode frequencies (ω_o and ω_m), vacuum optomechanical coupling rate, g_0, and scattering-limited optical quality factor, $Q_\mathrm{c,scat}$. To maximize $\gamma_\mathrm{OM} = 4g_0^2 n_\mathrm{c}/\kappa$, we attempted to maximize both g_0 and loaded Q-factor Q_c. Intrinsic quality factors of fabricated devices rarely get higher than $Q_\mathrm{c,i} = 10^6$ due to fabrication imperfections and optical absorption, while simulated $Q_\mathrm{c,scat}$ is generally high ($Q_\mathrm{c,scat} > 5 \times 10^6$) for a properly formed optical cavity. Therefore, we filtered out simulated geometries in which $Q_\mathrm{c,scat} < 2 \times 10^6$ to prevent radiative scattering from degrading Q_c in the fabricated device. After filtering out low Q designs, we assigned each design a fitness value simply given by $F \equiv -|g_0|$. We had 9 parameters over which we optimized F, which were d, h_i, w_i, h_o, w_o, $h_\mathrm{i,c}$, $w_\mathrm{i,c}$, $h_\mathrm{o,c}$, and $w_\mathrm{o,c}$. The parameters $h_\mathrm{i,c}$, $w_\mathrm{i,c}$, $h_\mathrm{o,c}$, and $w_\mathrm{o,c}$ are for the 'C'-shape holes in the center of the cavity. Parameters of the other cavity 'C'-shape holes between the mirror and the center on both sides were vaired quadratically with distance from the center holes. Note a, r and w were previously optimized for the large optical and mechanical bandgaps, and the thickness of the device layer, $t = 220$ nm, was fixed by the choice of substrate.

For a computationally expensive fitness function with a large parameter space, a good choice of optimization algorithm is the Nelder-Mead method [2]. Such a simplex search algorithm does not have smoothness requirements for the fitness function, making it quite resistant to simulation noise. A modern variant of this method is also implemented in fminsearch function of MATLAB. An optimization for quasi-2D OMC design is creates as follow:

1. To ensure realizable (ones we can fabricate) geometries are generated in a simulation, the parameter sets need to meet certain conditions. For example, $h_\mathrm{o} - h_\mathrm{i} \geq 60$ nm (55 nm for some of iterations) and $w_\mathrm{o}/2 - w_\mathrm{i}/2 \geq 60$ nm, where 60 nm is a conservative gap size we can realize with the limits of our device fabrication. Therefore, parameter sets are bounded for the generation of initial values and intermediate steps with the Nelder-Mead method.

2. Randomly generate an initial parameter set (d, h_i, w_i, h_o, w_o, $h_\mathrm{i,c}$, $w_\mathrm{i,c}$, $h_\mathrm{o,c}$, $w_\mathrm{o,c}$) within the bounds we set in step 1.

3. Run the optical simulation to determine the optical wavelengths (ω_o) of all the optical modes near 1550 nm with scattering-limited Q-factors larger than a threshold value (in practice only the fundamental mode we are interested in for most cases). If this fails, set $F = 0$ and go to step 6.

4. Scale all parameters except t, including a, r, and w, to move the optical mode with highest $Q_\mathrm{c,scat}$ to approximately 1550 nm.

5. Run the optical simulation again, in addition to the mechanical simulation, with scaled parameters, to determine ω_o, ω_m and g_0, and compute the fitness of the current scaled parameter set. If F did not change appreciably over the last few iterations, we reached a local minimum. Otherwise, we choose a new initial point by going to step 6.

6. Generate a new parameter set via the Nelder-Mead method and go to step 3.

By continually repeating the optimization algorithm, we mitigated the problem of converging on a local minimum. A visual representation of the search pattern is shown in supplementary figure S-1. We follow these steps until we have a design with a g_0 and $Q_\mathrm{c,scat}$ that we are satisfied with. The visual representation of supplementary figure S-1 is formed by ~ 5000 individual simulations (each individual simulation is defined after step 5 has finished successfully). We slice the multidimensional parameter space using $h_\mathrm{i,c}$ and $h_\mathrm{o,c}$ since the gap formed by these two parameters was where both mechanical displacement and optical field are most concentrated. Also, mechanical resonance ω_m highly depends on $h_\mathrm{i,c}$. Indeed, we noticed that most of the local minima lie on the line formed by $h_\mathrm{o,c} - h_\mathrm{i,c} = 60$ nm; this is because the intensity of optical field on the boundaries of the gap becomes stronger as the gap becomes narrower, hence the moving boundary was able contribute a larger amount of coupling due to higher overlap between optical and mechanical fields. This implied that if we could create a narrower gap in the realized devices we could get an even higher g_0. However, for current measurements, we chose a conservative gap value of $h_\mathrm{o,c} - h_\mathrm{i,c} \geq 60$ nm.

2. MEASUREMENT SETUP

The measurement setup used for device characterization is shown in Fig. S-2. A fiber-coupled, wavelength-tunable external cavity diode laser was used as the laser pump in all of our measurements. A small percentage of the laser output was sent to a wavemeter (λ-meter) for frequency stabilization. The laser was then passed through an initial

50 MHz-bandwidth tunable fiber Fabry-Perot filter (Micron Optics FFP-TF2) to reject laser phase noise at the mechanical frequency [3]. After this prefiltering, the light could be switched by 2×2 mechanical optical switches between two paths: (i) a balanced heterodyne detection path for performing spectroscopy of the cavity acoustic mode of the quasi-2D OMC cavity, and (ii) a photon counting path in which the laser could be modulated to create a train of high-extinction optical pulses and detection is performed using a single photon detector (SPD) and a time-correlated single photon counting (TCSPC) module.

For the balanced heterodyne path, a 90 : 10 beam-splitter divided the pump laser into local oscillator (LO, 90%, 0.5 - 1 mW) and signal (10%) beams. The LO was modulated by an electro-optic modulator (EOM) to generate a sideband at $\delta/2\pi = 50$ MHz above that of the acoustic mode frequency ($\omega_\mathrm{m} \approx 10$ GHz). This LO sideband is then selected by high-finesse tunable Fabry-Perot filter before recombining it with the signal. The signal beam is sent to a variable optical attenuator and optical circulator which directs it to a device under test in the dilution refrigerator. The reflected signal beam carrying mechanical noise sidebands at $\omega_\mathrm{l} \pm \omega_\mathrm{m}$ was recombined with the LO on a tunable variable optical coupler (VC), the outputs of which were sent to a balanced photodetector (BPD). The detected difference photocurrent contains a beat note corresponding to the acoustic cavity mode response near the LO detuning δ, chosen to lie within the detection bandwidth of the BPD.

For the photon counting path, the laser is prefiltered and routed to an electro-optic phase modulator (ϕ-EOM) which is driven at the mechanical frequency to generate optical sidebands for locking the pump-cancelling filters (on the detection side). The laser is then directed via 2×2 mechanical optical switches into a 'high-extinction' branch consisting of an acousto-optic modulator (AOM, 20 ns rise and fall time, 50 dB extinction) and a pair of Agiltron NS 1×1 high-speed switches (Ag., 100 ns rise time, 30 μs fall time, total of 36 dB extinction). The AOM and Ag. switches are driven by a digital delay generator to generate high-extinction-ratio optical pulses. The digital delay generator synchronizes the switching of the AOM and Ag. switches with the TCSPC module connected to SPD. The total optical extinction of the optical pulses is approximately 86 dB. After the 'high-extinction' branch, the laser light is also passed through the variable optical attenuator (VOA) and a circulator as in the balanced heterodyne path. The reflected signal beam carrying mechanical sidebands was routed to the detection side of the photon counting path. There, the light passes through three cascaded high-finesse tunable fiber Fabry-Perot filters (Micron Optics FFP-TF2) insulated from ambient light, and then sent to a SPD inside the dilution refrigerator.

The SPDs used in this work were amorphous WSi-based superconducting nanowire single-photon detectors developed in collaboration between the Jet Propulsion Laboratory and NIST. The SPDs were designed for a wavelength range $\lambda = 1520 - 1610$ nm, with maximum count rates as large as 10^7 counts per second (c.p.s.) [4]. The SPDs are mounted on the still stage of the dilution refrigerator at ~ 800 mK. Single-mode optical fibers are passed into the refrigerator through vacuum feedthroughs and coupled to the SPDs via a fiber sleeve attached to each SPD mount. The radio-frequency output of each SPD is amplified by a cold-amplifier mounted on the 50 K stage of the dilution refrigerator as well as a room-temperature amplifier, and read out by a triggered PicoQuant PicoHarp 300 time-correlated single photon counting module. After filtering out long-wavelength blackbody radiation inside the DR through a bandpass optical filter and isolating the input optical fiber from environmental light sources at room temperature, we observed SPD dark count rates as low as ~ 0.6 (c.p.s.) and a SPD quantum efficiency $\eta_\mathrm{SPD} \simeq 60\%$.

The tunable fiber Fabry-Perot filters used for both pre-filtering the pump and filtering the cavity have a bandwidth of 50 MHz, a free-spectral range of 20 GHz, and a tuning voltage of ≤ 18 V per free-spectral range. Each of the filters provides approximately 40 dB of pump suppression at 10 GHz offset compared to peak transmission; in total, the filters suppress the pump by > 100 dB. The three cascaded filter need to be regularly re-locked since they drift during measurement due to both thermal drift and acoustic disturbances in the environment, so they were placed inside a custom-built insulated housing to further improve stability. For the re-locking routine, we switch out of the 'high-extinction' (SW-2A,2B) branch and SPD branch (SW-5), as well as the branch which leads to the device under test (SW-3A, the other side connection not shown), to avoid sending large amount of power into the cavity and SPDs. We then drive a EOM (not shown) to generate large optical sidebands on the pump laser signal, one of which is aligned with the cavity resonance ($\omega_\mathrm{m}/2\pi$). This modulated signal is sent to the cascaded filters. To re-lock the filter chain, a sinusoidal voltage (0.5 V) was used to dither each filter while monitoring its transmission. The DC offsets of the dithering sinusoidal signal are then changed while reducing the sinusoidal voltage amplitude to maximize transmission of the desired sideband. After re-locking, the cavity, SPD and 'high-extinction' branches are switched back into the optical train, and a new round of measurements can be performed. The total filter transmission was recorded at the end of a re-locking routine and subsequent measurement run, and the previous measurement run was discarded if the transmission shifted by more than a few percent.

3. OPTICAL COUPLING TO DEVICES

The device sample is mounted at the mixing chamber of the DR, with a fiber-to-chip coupling realized by an end-fire coupling scheme with an anti-reflection-coated tapered lensed fiber [5]. The tapered lensed fiber was placed on a position-encoded piezo xyz-stage in close proximity to the device chip. After cooling the experiment from room temperature to ~ 10 mK, we optimize the fiber tip position relative to a tapered 1D coupling waveguide on the device layer by monitoring the reflected optical power on a slow photodetector.

The design of the tapered 1D coupling waveguide is similar to those found in Refs. [6] and [5]. The tip of the waveguide was designed to mode match the field of the waist of the lensed fiber. The major distinction for the 2D case was that the other side of the tapered waveguide coupler was also designed to mode match to the line-defect waveguide in the 2D region as shown in Fig. S-3a. The mirror in the 2D line-defect waveguide region was introduced gradually, to avoid excess scattering in this region. The shape of the center of the line-defect waveguide was slowly changed from a geometry that provides no photonic bandgap, over a number of periods n_{trans}, to the 'C' shape which provides a photonic bandgap. Following n_{trans}, there were a variable number of mirror periods n_{mirr}. Reducing n_{mirr} made a partially transparent mirror which serves as one side of the cavity's end-mirrors. Thus, a controllable amount of the incident light was permitted to leak through to the cavity region while both of the mirror and defect region of the cavity were highly reflective at frequencies far from resonance.

Supplementary Figure S-3b shows the broadband reflection spectrum of the optimized coupler, calculated by a finite-difference-time-domain simulation [7]. The amplitude and free spectral range of fringes in the spectrum are consistent with a low finesse Fabry-Pérot cavity formed by weak waveguide-air-interface reflection $R \approx 1.6\%$ and the near unity reflectivity of the quasi-2D OMC cavity mirror. A single-pass coupling efficiency η_{cpl} is estimated from the broadband reflection spectrum shown in Fig.S-3c. The actual measured single-pass efficiencies were $\eta_{\mathrm{cpl}} \approx 60.7\%$ for a zero-shield device and $\eta_{\mathrm{cpl}} \approx 59.7\%$ for an eight-shield device. The difference between simulations and measurements is attributed to slight fabrication offsets—a small offset on the scale of several nanometers for the width of tapered 1D coupling waveguide may cause significant mode mismatch on both sides.

4. ACOUSTIC SHIELD

To minimize mechanical clamping losses, the quasi-2D OMC was surrounded by an additional shield structure designed to have a complete phononic bandgap at the quasi-2D OMC cavity mode frequency [8, 9]. Geometrically, the structure consists of a square lattice of cross-shaped holes, or equivalently, an array of squares connected to each other via narrow bridges. We call this the 'cross-shield' or 'cross-structure'. The phononic bandgap in the cross-structure comes from the frequency separation between the normal-mode resonances of the individual squares and the lower frequency 'acoustic bands'. These acoustic bands are strongly dependent on the width of the connecting narrow bridges, a_{c}-h_{c}, where a_{c} is lattice constant and h_c is the height of cross holes as indicated in Fig S-4a.

We analyzed SEM images of fabricated structures to provide parameters for our FEM simulation. For exampe, we included filleting of the inner and outer corners (r_1 and r_2 in Fig. S-4a) in our simulation, arising from the technical limitations of our nanofabrication methods. The silicon device layer used in the simulations is 220 nm in thickness, with mass density of 2329 kg/m^2, and anisotropic elasticity matrix $(C_{11}, C_{12}, C_{44}) = (166, 64, 80)$ GPa , assuming a [100] crystallographic orientation along the x-axis. As shown in Fig. S-4b, a bandgap > 4 GHz centered around ~ 10 GHz is realized through tuning of the cross lattice constant a_{c}, cross height h_{c} and width w_{c}.

5. ELECTROMAGNETICALLY INDUCED TRANSPARENCY MECHANICAL SPECTROSCOPY

Electromagnetically induced transparency (EIT) in optomechanical systems [10, 11] allows for a spectral measurement of the mechanical response via observation of a transparency window in the optical reflection spectrum. A pump laser tone at ω_{l} is amplitude modulated at frequency $\Delta_{\mathrm{p}}/2\pi$ to generate a weak probe tone at $\omega_{\mathrm{s},\pm} = \omega_{\mathrm{l}} \pm \Delta_{\mathrm{p}}$. If the pump-cavity detuning is fixed on either the red- or blue-side of the optical cavity ($\Delta \equiv \omega_{\mathrm{c}} - \omega_{\mathrm{l}} = \pm\omega_{\mathrm{m}}$), the optical susceptibility of the cavity strongly suppresses one of the probe sidebands (at $\omega_{\mathrm{s},\mp}$) and only the other probe sideband will have an appreciable intracavity population. For a red-detuned pump, the interaction of the pump tone and mechanics with the probe sideband yields a reflection coefficienct $r(\Delta, \delta)$ for the probe which contains a transparency window having a width on the scale of the mechanical mode linewidth:

$$r(\Delta, \delta) = 1 - \frac{\kappa_{\mathrm{e}}}{\kappa/2 + i(\Delta - (\delta + \omega_{\mathrm{m}})) + \frac{|G|^2}{-i\delta + \gamma_{\mathrm{i}}/2}}, \tag{S-1}$$

where we have defined $\delta \equiv \Delta_\mathrm{p} - \omega_\mathrm{m}$ and $G \equiv g_0\sqrt{n_\mathrm{c}}$. We measure the reflection amplitude $R = |r|^2$ by driving an EOM weakly to generate a probe tone and observing the count rates of sideband-scattered probe photons. The pump is locked at $\Delta = +\omega_\mathrm{m}$ and the cascaded filter stack is locked to the cavity frequency. The RF modulation power is chosen to generate a sideband intracavity photon number ($n_{\mathrm{c},+}$) much smaller than the carrier photon number (n_c), $n_{\mathrm{c},+} \ll n_\mathrm{c}$.

6. THERMAL CONDUCTANCE SIMULATIONS OF 1D AND 2D OMC CAVITIES

Here we utilize FEM simulations to model the impact of geometry on the thermal conductance of different OMC cavities at millikelvin temperatures. Specifically our approach is as follows. We take previous measurements of a 1D nanobeam OMC cavity and compare it to the results of simulations of a similar 1D OMC cavity geometry with variable material properties. We then find what scaling of the material properties allows us to match experiment to simulation for the 1D OMC cavity. Using these same scaled values of the material properties we then perform simulations of the quasi-2D OMC cavity. Closing the loop, we find that the simulated values of the hot bath temperature are in correspondence with the measured values for the quasi-2D cavity. This would indicate that a simple geometric difference in the connectivity of the 1D and 2D cavities to the external chip bath can explain the lower measured hot bath occupancy for the quasi-2D OMC cavity, validating our original design concept.

Under steady state conditions, the power flow from the hot bath into the DR bath, P_th, is equal to the power flow into the hot bath due to optical absorption. Here, we have implicitly assumed no other sources of heating other than optical absorption and that the hot bath loses energy via coupling to phonons which radiate into the chip bath at the periphery of the device. Also assuming the optical absorption process is linear, we find that the power flow into the hot bath is a fraction η_abs of the total input optical power, such that $P_\mathrm{th} = \eta_\mathrm{abs} P_\mathrm{in} \propto n_\mathrm{c}$ (we ignore n_wg here for simplicity).

For the temperature range considered in this work (where phonon transport is ballistic), the lattice thermal conductivity scales as a power law of the phonon bath temperature [12, 13]. We thus define the thermal conductance from the center of OMC cavity to the DR bath of both the 1D ($C_\mathrm{th,1D}$) and quasi-2D geometries ($C_\mathrm{th,2D}$) such that $C_\mathrm{th} \propto (T_\mathrm{p})^\alpha$. The exponent α is equal to the effective number of spatial dimensions d of the geometry. The hot bath is assumed to thermalize at an effective temperature T_p and to radiate energy (lattice phonons) into the periphery of the cavity as a black body such that the power lost out of the hot bath goes as $(T_\mathrm{p})^{\alpha+1}$. We can thus write a simple model for the thermal conductance between the hot bath and the periphery of the cavity (T_0),

$$P_\mathrm{th} = C_\mathrm{th}\Delta T \approx C_\mathrm{th} T_\mathrm{p}, \tag{S-2}$$

where $\Delta T = T_\mathrm{p} - T_0$ and in the range of measured n_p ($n_\mathrm{p} > 0.5, T_\mathrm{p} > 400$ mK) $\Delta T \approx T_\mathrm{p}$ since $T_0 \ll T_\mathrm{p}$. We define C_th as $C_\mathrm{th} = \epsilon (T_\mathrm{p})^\alpha$, where ϵ depends on the geometry of the cavity and its material properties, which allows us to write,

$$P_\mathrm{th} = \epsilon T_\mathrm{p}^{\alpha+1} = \eta_\mathrm{abs} P_\mathrm{in} \propto n_\mathrm{c}. \tag{S-3}$$

The power law exponent α in the thermal conductance model is estimated to be $\alpha_0 \approx 2.3$ from the measured data (see Supplementary Figure S-5a). This is consistent with a Si slab of thickness $t = 220$ nm that has an approximately 2D phonon density of states for acoustic modes of frequency in the vicinity of the upper band-edge of the phononic bandgap of the quasi-2D snowflake structure ($\omega/2\pi \gtrsim 10$ GHz).

Assuming a thermal conductivity for the Si slab which is proportional to $(T_\mathrm{p})^{\alpha_0}$, FEM simulations were performed on both the 1D nanobeam and the quasi-2D snowflake OMC cavity geometries. As a thermal excitation source we placed a heating source in the center of both OMC cavities with size corresponding to that of the optical mode volume of the cavity mode. The average temperatures within the optical mode volume ($T_{\mathrm{p},(1,2)\mathrm{D}}$) was then calculated versus intra-cavity photons (n_c) for the 1D nanobeam cavity. We adjusted the material properties (thermal conductivity and absorption coefficient) in order to match the simulated curve to the measured data of the 1D nanobeam cavity from Ref. [14]. Finally, we used these adjusted material properties to simulate the quasi-2D cavity. All measured and simulated curves are plotted in Fig. S-5a. We also plot the temperature profile of the 1D and quasi-2D OMC cavities at $n_\mathrm{c} = 100$ in Figs. S-5b and S-5c, respectively.

By comparing the simulated curves in Supplementary Figure S-5a, we estimate that the thermal conductance of the quasi-2D and 1D structure has a ratio of $\epsilon_\mathrm{2D}/\epsilon_\mathrm{1D} \approx 42$. For the same optical pump power applied to the 1D and quasi-2D OMC cavities we have that $n_\mathrm{c,1D} = n_\mathrm{c,2D}$ and $P_\mathrm{th,1D} = P_\mathrm{th,2D}$. This yields the relation between thermal conductance and acoustic mode occupancy for the two cavity geometries,

$$\epsilon_{1\mathrm{D}}\left(\frac{\hbar\omega_{\mathrm{m,1D}}n_{\mathrm{p,1D}}}{k_B}\right)^{\alpha_0+1}=\epsilon_{2\mathrm{D}}\left(\frac{\hbar\omega_{\mathrm{m,2D}}n_{\mathrm{p,2D}}}{k_B}\right)^{\alpha_0+1}, \tag{S-4}$$

where we have assumed $n_{\mathrm{p}} \approx k_B T_{\mathrm{p}}/\hbar\omega_{\mathrm{m}}$ in rewriting the bath temperatures in each cavity in terms of the bath occupancy at the acoustic cavity mode frequency. Considering that the acoustic mode of the quasi-2D OMC is at frequency $\omega_{\mathrm{m,2D}}/2\pi \approx 10.27$ GHz while that of the 1D resonator is at half this frequency at $\omega_{\mathrm{m,1D}}/2\pi \approx 5$ GHz, we can write for the ratio of the effective bath occupancies in the two cavities,

$$\frac{n_{\mathrm{p,1D}}}{n_{\mathrm{p,2D}}} \approx 2\left(\frac{\epsilon_{2\mathrm{D}}}{\epsilon_{1\mathrm{D}}}\right)^{1/(\alpha_0+1)}=6.2. \tag{S-5}$$

This simulated ratio is in good agreement with the measured ratio of the phonon bath occupancy of the 1D nanobeam OMC cavity in Ref. [14] and the quasi-2D OMC cavity of this work, $n_{\mathrm{p,1D}}/n_{\mathrm{p,2D}} = 7.94/1.1 \approx 7.2$.

7. FITTING OF THE MECHANICAL LINEWIDTH DEPENDENCE ON INTRACAVITY PHOTON NUMBER

In order to fit the mechanical linewidth dependence on intracavity photo number n_{c}, we see that the linewidth can be broken into three distinct regimes: (i) at the lowest powers ($n_{\mathrm{c}} \lesssim 10$) the linewidth saturates to a constant value given by γ_ϕ (γ_0 is entirely negligible on this scale), (ii) a low-power regime ($100 < n_{\mathrm{c}} < n_{\mathrm{th}} \approx 1000$) with a relatively strong dependence of linewidth on optical power, and (iii) a high-power regime ($n_{\mathrm{c}} > n_{\mathrm{th}} \approx 1000$) with a second, weaker dependence of linewidth on optical power, where n_{th} is the threshold for n_{c} used between (ii) and (iii) in the fitting. In order to capture this behavior in a single fitting function we have defined,

$$\gamma = G(\gamma_\phi + \Gamma_{\mathrm{L}} n_{\mathrm{c}}^{\xi_{\mathrm{L}}}) + (1-G)(\gamma_{\phi,\mathrm{H}} + \Gamma_{\mathrm{H}} n_{\mathrm{c}}^{\xi_{\mathrm{H}}}), \tag{S-6}$$

with $G = 1/(1+n_{\mathrm{c}}/n_{\mathrm{th}})^2$ a saturation parameter which is used to smoothly transition between the low and high power regimes. In this model, n_{th}, Γ_{L}, Γ_{H}, ξ_{L}, ξ_{H}, γ_ϕ and $\gamma_{\phi,\mathrm{H}}$ are all fitting parameters. The best fit gives $n_{\mathrm{th}} \approx 1000$, $\Gamma_{\mathrm{L}} = 1.1$ kHz, $\Gamma_{\mathrm{H}} = 9.01$ kHz, $\xi_{\mathrm{L}} = 0.61$, $\xi_{\mathrm{H}} = 0.29$, $\gamma_\phi = 14.54$ kHz and $\gamma_{\phi,\mathrm{H}} = 23.91$ kHz, corresponding to $\gamma/2\pi = \gamma_\phi/2\pi + (1.1\ \mathrm{kHz}) \times n_{\mathrm{c}}^{0.61}$, with $\gamma_\phi/2\pi = 14.54$ kHz in the low-power regime and $\gamma/2\pi = 23.91\ \mathrm{kHz} + (9.01\ \mathrm{kHz}) \times n_{\mathrm{c}}^{0.29}$ in the high power regime. At lower powers we find a power-law scaling and overall magnitude of damping of the breathing mode of the quasi-2D OMC cavity which is close to that for the 1D nanobeam cavities of Ref. [14]. In the high-power regime we find a fit with a power-law exponent that is approximately half of that in the low-power regime. The physical reason for this change in power-law is still under exploration.

8. MODELING OF ADDITIONAL HEATING IN THE COUPLING WAVEGUIDE

In order to better understand the source of the modifies back-action cooling curves measured for the quasi-2D OMC cavities of this work, we performed optical FEM simulations on the full device, including the OMC cavity, 1D coupling waveguide and 2D line-defect waveguide. As mentioned, the coupling waveguide in quasi-2D devices was designed to be physically connected to one end of the OMC cavity instead of evanescently coupled to the OMC cavity as in 1D nanobeam OMC devices [14, 15]. We find below that due to the weak reflectivity of the air-waveguide interface, a weak cavity is formed in the waveguides. As a result, there are two major areas of optical absorption found to be contributing to the hot bath: (i) intra-cavity photons n_{c} coupled into the OMC cavity and (ii) photons being coupled into the weak cavity. In such a scenario, the occupation of the hot bath n_{p} can depend on both intra-cavity photon number n_{c} and the input laser power P_{in} in the coupling waveguide. Here we use an effective waveguide phonon number n_{wg} ($n_{\mathrm{wg}} \propto P_{\mathrm{in}}$) to represent the contribution from photons in the weak cavity of the coupling waveguide.

In order to estimate the effect of the optical absorption in the coupling waveguide in comparison to that in the cavity we performed FEM simulations using a geometry which was tuned to approximately the same optical properties as the eight-shield device used for characterizing the hot bath in the main text ($\kappa/2\pi = 1.187$ GHz, $\kappa_{\mathrm{i}} = 1.006$ GHz). For optical laser detuning $\Delta = 0$, shown in Fig. S-6a, a large portion of the input photons that couple into the coupling waveguide are coupled into OMC cavity, with the electric field energy in the OMC cavity one order-of-magnitude higher than in the weak cavity region ($n_{\mathrm{c}} \gg n_{\mathrm{wg}}$). For optical laser detuning of $\Delta = 10$ GHz $\approx \omega_{\mathrm{m}}$, shown in

Figure S-6b, a much smaller portion of photons coupled into the coupling waveguides are eventually coupled into OMC cavity due to the large cavity reflection due to the cavity detuning ($\kappa \ll \omega_{\mathrm{m}}$). In this case the electric field energy in the OMC cavity is only a few percent of the energy in the weak cavity region ($n_{\mathrm{c}} \ll n_{\mathrm{wg}}$). Taken together, these two simulations provide strong evidence for the conclusion in the main text that: (i) for on-resonance optical pumping one can ignore the effects of n_{wg}, and (ii) for back-action cooling with laser detuning $\Delta = \omega$, the optical absorption in the coupling waveguide directly proportional to P_{in} should add significant heating of the acoustic mode of the quasi-2D OMC cavity.

9. MODE THERMALIZATION MEASUREMENTS

To measure the true base temperature T_0 of the quasi-2D OMC cavity devices on the chip we used a low-power ($n_{\mathrm{c}} = 9.9$) laser pump and studied a device with relatively high mechanical damping $\gamma_0 = 21.8$ kHz (zero-shield device, $Q_{\mathrm{m}} = 4.69 \times 10^5$) so that data integration time was minimized. With relatively high mechanical damping, the mechanical mode quickly thermalizes to its base temperature between subsequent incident optical pulses so that we could use a rapid measurement repetition rate $1/\tau_{\mathrm{per}}$ ($\tau_{\mathrm{per}} = \tau_{\mathrm{pulse}} + \tau_{\mathrm{off}} \gg \gamma_0^{-1}$). The initial mode occupancy during the pulse then approximately corresponds to the base bath occupancy n_0. In order to remove initial heating from the optical pulse, we fit the entire curve of the measured phonon occupancy throughout the pulse using a phenomenological model of the dynamics of the hot bath and the heating and damping of the acoustic mode [14], and extrapolated the fit back to the start of pulse to estimate n_0.

Supplementary Fig.S-7 shows the measured acoustic mode occupancy versus time within the optical pulse of the quasi-2D OMC cavity. Theoretical curves from the phenomenological model for $T_0 = 11$ mK (red), 31 mK (yellow), 63 mK (blue), 95 mK (green), 129 mK (cyan) are plotted for reference. From these curves the base temperature of the surface of the Si chip is estimated to be $T_0 \lesssim 63$ mK, corresponding to a base mode occupation of $n_0 \lesssim 4 \times 10^{-4}$.

10. PHONON MEDIATED QUANTUM STATE TRANSDUCTION BETWEEN MICROWAVE AND OPTICAL PHOTONS

In this section we provide an example of coherent quantum transfer protocol that demonstrates the significance of quantum cooperativity – phonon mediated bi-directional microwave-to-optical transduction [16–21]. In particular, we present the analysis of a proposed piezo-optomechanical quantum transducer and study how the transduction efficiency and signal-to-noise ratio depend on C_{eff}. Figure S-8 shows schematically the different elements of the proposed transducer. In this scheme, a microwave frequency mechanical mode of an optomechanical cavity is coupled resonantly to a tunable superconducting microwave resonator (frequency (ω_μ) through the piezoelectric effect with rate J_{pa}. The optical mode (frequency ω_{o}) of the optomechanical cavity is coupled to the input fiber with coupling rate κ_{oe}, and it suffers from intrinsic loss to environment with rate κ_{oi}. The superconducting microwave resonator is coupled to a transmission line with rate $\kappa_{\mu\mathrm{e}}$ and it has intrinsic loss rate $\kappa_{\mu\mathrm{i}}$ to the environment.

The system Hamiltonian for this open quantum system is given by,

$$H = H_0 + H_{\mathrm{int}} + H_{\mathrm{drive}} + H_{\mathrm{signal}} + H_{\mathrm{noise}}, \tag{S-7}$$

$$H_0 = \hbar(\omega_{\mathrm{o}} - \frac{\kappa_{\mathrm{o}}}{2}i)\hat{a}^\dagger\hat{a} + \hbar(\omega_{\mathrm{m}} - \frac{\gamma}{2}i)\hat{b}^\dagger\hat{b} + \hbar(\omega_\mu - \frac{\kappa_\mu}{2}i)\hat{c}^\dagger\hat{c}, \tag{S-8}$$

$$H_{\mathrm{int}} = \hbar g_0(\hat{a}^\dagger\hat{b} + \hat{a}\hat{b}^\dagger) + \hbar j_0(\hat{c}^\dagger\hat{b} + \hat{c}\hat{b}^\dagger), \tag{S-9}$$

$$H_{\mathrm{drive}} = -\hbar\sqrt{\kappa_{\mathrm{oe}}}i(\alpha^*_{\mathrm{in}}(t)\hat{a} + \alpha_{\mathrm{in}}(t)\hat{a}^\dagger), \tag{S-10}$$

$$H_{\mathrm{signal}} = -\hbar\sqrt{\kappa_{\mathrm{oe}}}i(\hat{a}^\dagger_{\mathrm{in}}\hat{a} + \hat{a}_{\mathrm{in}}\hat{a}^\dagger) - \hbar\sqrt{\kappa_{\mu\mathrm{e}}}i(\hat{c}^\dagger_{\mathrm{in}}\hat{c} + \hat{c}_{\mathrm{in}}\hat{c}^\dagger), \tag{S-11}$$

$$H_{\mathrm{noise}} = -\hbar\sqrt{\kappa_{\mathrm{oi}}}i(\hat{a}^\dagger_{\mathrm{n}}\hat{a} + \hat{a}_{\mathrm{n}}\hat{a}^\dagger) - \hbar\sqrt{\gamma}i(\hat{b}^\dagger_{\mathrm{n}}\hat{b} + \hat{b}_{\mathrm{n}}\hat{b}^\dagger) - \hbar\sqrt{\kappa_{\mu\mathrm{i}}}i(\hat{c}^\dagger_{\mathrm{n}}\hat{c} + \hat{c}_{\mathrm{n}}\hat{c}^\dagger). \tag{S-12}$$

where $\alpha_{\mathrm{in}}(t) = \alpha_0 \exp(-i\omega_{\mathrm{d}}t)$ is the optical pumping field at ω_{d}. Here, the intrinsic loss channels introduce environmental noise ($\hat{a}_{\mathrm{n}}$,$\hat{b}_{\mathrm{n}}$,$\hat{c}_{\mathrm{n}}$) into the system, and signals ($\hat{a}_{\mathrm{in,out}}$ and $\hat{c}_{\mathrm{in,out}}$) are coupled into and out of the transducer via external coupling channels to the optical fiber port (left side in Fig. S-8) and the superconducting transmission line (right side in Fig. S-8).

These equations can be solved in the linearized regime assuming a red-detuned drive and sideband resolution. The transducer conversion efficiency is found to be limited by the internal mechanical decay, while the transducer noise is set by thermal mechanical noise and noise from each of the external ports. For the details of the treatment see Refs. [22] and [23]. The conversion number efficiency is the same for either conversion direction, and is given by $\eta_{\mathrm{o}\rightleftarrows\mu}$.

The signal-to-noise ratio when considering an input signal corresponding to a single photon is related to the inverse of an added noise occupancy number. For optical-to-microwave conversion the appropriate single photon signal-to-noise is $\mathrm{SNR}_{\mathrm{o}\rightarrow\mu}$, whereas for microwave-to-optical it is $\mathrm{SNR}_{\mathrm{o}\leftarrow\mu}$. The solutions for conversion number efficiency and single photon signal-to-noise ratios are:

$$\eta_{\mathrm{o}\rightleftarrows\mu} = \left| \left| \frac{1}{D} \right|^2 \sqrt{\kappa_{\mathrm{oe}}\kappa_{\mu\mathrm{e}}} GJ \right|^2, \tag{S-13}$$

$$\mathrm{SNR}_{\mathrm{o}\leftarrow\mu} = \frac{\kappa_{\mu\mathrm{e}} G^2 J^2}{\kappa_{\mathrm{oi}} \left|(\omega_{\mathrm{m}} - \omega - \frac{\gamma}{2}i)(\omega_\mu - \omega - \frac{\kappa_\mu}{2}i) - J^2\right|^2 \bar{n}_{\mathrm{ob}} + \gamma G^2 \left|\omega_\mu - \omega - \frac{\kappa_\mu}{2}i\right|^2 \bar{n}_{\mathrm{mb}} + G^2 J^2 \kappa_{\mu\mathrm{i}} \bar{n}_{\mu\mathrm{b}}}, \tag{S-14}$$

$$\mathrm{SNR}_{\mathrm{o}\rightarrow\mu} = \frac{\kappa_{\mathrm{oe}} G^2 J^2}{\kappa_{\mu\mathrm{i}} \left|(\omega_{\mathrm{m}} - \omega - \frac{\gamma}{2}i)(\omega_{\mathrm{o}} - \omega - \frac{\kappa_{\mathrm{o}}}{2}i) - G^2\right|^2 \bar{n}_{\mu\mathrm{b}} + \gamma J^2 \left|\Delta_{\mathrm{o}} - \omega - \frac{\kappa_{\mathrm{o}}}{2}i\right|^2 \bar{n}_{\mathrm{mb}} + G^2 J^2 \kappa_{\mathrm{oi}} \bar{n}_{\mathrm{ob}}}, \tag{S-15}$$

with

$$D \equiv \left((\Delta_{\mathrm{o}} - \omega - \frac{\kappa_{\mathrm{o}}}{2}i)(\omega_{\mathrm{m}} - \omega - \frac{\Gamma}{2}i)(\omega_\mu - \omega - \frac{\kappa_\mu}{2}i) - G^2(\omega_\mu - \omega - \frac{\kappa_\mu}{2}i) - J^2(\Delta_{\mathrm{o}} - \omega - \frac{\kappa_{\mathrm{o}}}{2}i) \right)^{-1}. \tag{S-16}$$

Here the thermal noise bath occupancies are labelled as $\bar{n}_{\mathrm{mb}}$, $\bar{n}_{\mathrm{ob}}$, and $\bar{n}_{\mu\mathrm{b}}$, corresponding to the mechanical thermal bath, the optical cavity thermal bath, and the microwave electrical resonator thermal bath, respectively. Note that the conversion efficiency can be maximized with respect to the frequency ω by looking for the extrema points of D assuming the resonant condition, $\Delta_{\mathrm{o}} \equiv (\omega_{\mathrm{o}} - \omega_{\mathrm{d}}) = \omega = \omega_{\mathrm{m}} = \omega_\mu$.

To highlight the importance of quantum cooperativity for optomechanical and piezoelectric interactions we can further simplify equations for $\mathrm{SNR}_{\mathrm{o}\leftarrow\mu}$ and $\mathrm{SNR}_{\mathrm{o}\rightarrow\mu}$ at the maximum conversion efficiency points,

$$\begin{aligned}\mathrm{SNR}_{\mathrm{o}\leftarrow\mu} &= \frac{\kappa_{\mu\mathrm{e}} J^2}{\gamma A \bar{n}_{\mathrm{mb}}} \\ &= \frac{\kappa_{\mu\mathrm{e}}\kappa_\mu}{4A} C^{\mathrm{eff}}_{\mu,\mathrm{m}},\end{aligned} \tag{S-17}$$

$$\begin{aligned}\mathrm{SNR}_{\mathrm{o}\rightarrow\mu} &= \frac{\kappa_{\mathrm{oe}} G^2}{\gamma B \bar{n}_{\mathrm{mb}}} \\ &= \frac{\kappa_{\mathrm{oe}}\kappa_{\mathrm{o}}}{4B} C^{\mathrm{eff}}_{\mathrm{o,m}}\end{aligned} \tag{S-18}$$

where the quantum cooperativities are defined as $C^{\mathrm{eff}}_{\mu,\mathrm{m}} = C_{\mu,\mathrm{m}}/\bar{n}_{\mathrm{mb}}$ and $C^{\mathrm{eff}}_{\mathrm{o,m}} = C_{\mathrm{om}}/\bar{n}_{\mathrm{mb}}$, with standard cooperativities at the microwave and optical ports given as $C_{\mu,\mathrm{m}} = 4J^2/\gamma\kappa_\mu$ and $C_{\mathrm{om}} = 4G^2/\gamma\kappa_{\mathrm{o}}$, respectively. In the above equations, $A \equiv \left|\omega_\mu - \omega - \frac{\kappa_\mu}{2}i\right|^2$ and $B \equiv \left|\Delta_{\mathrm{o}} - \omega - \frac{\kappa_{\mathrm{o}}}{2}i\right|^2$, and we have dropped terms related to $\bar{n}_{\mu\mathrm{b}}$ and $\bar{n}_{\mathrm{ob}}$ since they are very small in the system discussed here (milliKelvin temperatures). We have also neglected spontaneous scattering noise (quantum noise) in the optomechanical interaction [22] due to the very large sideband ratio that we have in our 2D OMC system. From these simple relations we can see the importance of the quantum cooperativity for quantum transduction applications. In order to transduce single photons with SNR greater than unity one needs $C^{\mathrm{eff}}_{\mu,\mathrm{m}}$ and $C^{\mathrm{eff}}_{\mathrm{o,m}}$ to be larger than unity (the pre-coefficients in Eqs. (S-17) and (S-18) are always less than or equal to unity, depending on the level of overcoupling to the optical and microwave external lines).

[1] Meenehan, S. M. *et al.* Pulsed Excitation Dynamics of an Optomechanical Crystal Resonator near Its Quantum Ground State of Motion. *Physical Review X* **5**, 041002 (2015).

[2] Nelder, J. A. & Mead, R. A simplex method for function minimization. *The Computer Journal* **7**, 308–313 (1965).

[3] Meenehan, M. *et al.* Pulsed excitation dynamics of an optomechanical crystal resonator near its quantum ground-state of motion. *arXiv* **1503**, 1–16 (2015). 1503.05135.

[4] Marsili, F. *et al.* Detecting single infrared photons with 93efficiency. *Nature Photon.* **7**, 210–214 (2013).

[5] Meenehan, S. M. *et al.* Silicon optomechanical crystal resonator at millikelvin temperatures. *Phys. Rev. A* **90**, 011803 (2014).

[6] Cohen, J. D., Meenehan, S. M. & Painter, O. Optical coupling to nanoscale optomechanical cavities for near quantum-limited motion transduction. *Opt. Express* **21**, 11227–11236 (2013).

[7] Lumerical Solutions Inc. http://www.lumerical.com/tcad-products/fdtd/.

[8] Safavi-Naeini, A. H. & Painter, O. Design of optomechanical cavities and waveguides on a simultaneous bandgap phononic-photonic crystal slab. *Opt. Express* **18**, 14926–14943 (2010).

[9] Chan, J., Safavi-Naeini, A. H., Hill, J. T., Meenehan, S. & Painter, O. Optimized optomechanical crystal cavity with acoustic radiation shield. *Applied Physics Letters* **101** (2012). 1206.2099.

[10] Safavi-Naeini, A. H. *et al.* Electromagnetically induced transparency and slow light with optomechanics. *Nature* **472**, 69–73 (2011).

[11] Weis, S. *et al.* Optomechanically induced transparency. *Science* **330**, 1520–1523 (2010).

[12] Holland, M. G. Analysis of lattice thermal conductivity. *Phys. Rev.* **132**, 2461 (1963).

[13] Callaway, J. Model for lattice thermal conductivity at low temperatures. *Phys. Rev.* **113**, 1046–1051 (1959).

[14] MacCabe, G. S. *et al.* Phononic bandgap nano-acoustic cavity with ultralong phonon lifetime. *Preprint at https://arxiv.org/abs/1901.04129* (2019).

[15] Meenehan, S. M. *Cavity Optomechanics at Millikelvin Temperatures*. Ph.D. thesis (2015).

[16] Bochmann, J., Vainsencher, A., Awschalom, D. D. & Cleland, A. N. Nanomechanical coupling between microwave and optical photons. *Nature Physics* **9**, 712–716 (2013).

[17] Andrews, R. W. *et al.* Bidirectional and efficient conversion between microwave and optical light. *Nature Physics* **10**, 321–326 (2014).

[18] Balram, K. C., Davanço, M. I., Song, J. D. & Srinivasan, K. Coherent coupling between radiofrequency, optical and acoustic waves in piezo-optomechanical circuits. *Nature photonics* **10**, 346 (2016).

[19] Higginbotham, A. *et al.* Harnessing electro-optic correlations in an efficient mechanical converter. *Nature Physics* **14**, 1038 (2018).

[20] Bienfait, A. *et al.* Phonon-mediated quantum state transfer and remote qubit entanglement. *Science* **364**, 368–371 (2019).

[21] Lauk, N. *et al.* Perspectives on quantum transduction. *arXiv preprint arXiv:1910.04821* (2019).

[22] Hill, J. T., Safavi-Naeini, A. H., Chan, J. & Painter, O. Coherent optical wavelength conversion via cavity optomechanics. *Nature Commun.* **3**, 1196 (2012).

[23] Luo, J. *Integrating Quantum Optical and Superconducting Circuits with Quantum Acoustics for Scalable Quantum Network and Computation*. Ph.D. thesis, California Institute of Technology (2019).
